# Mindful minds: How group identity shapes brain and behavior in social decision-making

**DOI:** 10.3758/s13415-026-01425-1

**Published:** 2026-04-17

**Authors:** Maartje M. A. Overhaus, Mariët van Buuren, Paul A. M. van Lange, Lydia Krabbendam

**Affiliations:** 1 Department of Clinical-, Neuro-, and Developmental Psychology, Faculty of Behavioral and Movement Sciences, Vrije Universiteit Amsterdam, Van Der Boechorststraat 7, 1081 BT Amsterdam, The Netherlands; 2 Experimental and Applied Psychology, Vrije Universiteit Amsterdam, Van Der Boechorststraat 7, 1081 BT Amsterdam, The Netherlands; 3 Institute for Brain and Behavior Amsterdam, Vrije Universiteit Amsterdam, Van Der Boechorststraat 7, 1081 BT Amsterdam, the Netherlands

**Keywords:** Social mindfulness, Group identity, Political ideology, FMRI, Decision making

## Abstract

Social mindfulness is a form of prosocial behavior that requires limited effort and considers other’s needs and desires. But does this consideration persist when the other person supports opposing political ideologies? Using the social mindfulness (SoMi) paradigm with 45 adults holding strong pro- or anti-refugee stances, the current functional magnetic resonance imaging study investigates how group identity influences socially mindful behavior and its neural underpinnings. Consistent with prior research, individuals were more socially mindful toward ingroup members than toward outgroup members or unclassified others. This behavior engaged regions associated with mentalizing, decision-making and reward processing, including the temporoparietal junction, dorsal medial prefrontal cortex, dorsolateral prefrontal cortex, middle temporal gyrus, and orbitofrontal cortex. No neural differences emerged during socially mindful decision-making between ingroup and outgroup members or ingroup members and unclassified others. Interestingly, socially mindful decision-making for outgroup members, compared with ingroup members, elicited heightened activation in the right anterior insula, possibly reflecting the social and emotional significance of outgroup interactions resulting from experienced norm violation. Additionally, such decisions for outgroup members, as opposed to unclassified others, were associated with increased activation in the dorsal anterior cingulate cortex (dACC). Given the dACC’s role in cognitive control during complex decision-making, this finding suggests that individuals exerted more effort to align their behavior with prosocial norms, even when these conflicted with self-interest. Overall, this study highlights how shared group identity shapes social mindfulness, reflecting both the emotional processing of outgroup interactions and the mental effort required to maintain prosocial norms in intergroup contexts.

**Supplementary Information:**

The online version contains supplementary material available at 10.3758/s13415-026-01425-1.

## Introduction

Picture yourself on a coffee break at work with colleagues where someone has brought a tray of cookies. You notice that while there are still plenty of oatmeal cookies, only one chocolate chip cookie remains. You opt for an oatmeal cookie, leaving the last chocolate chip cookie for the colleague behind you. This small gesture might seem trivial but could be greatly appreciated by your colleague and could even strengthen your relationship. But would you still leave the chocolate chip cookie so willingly if that colleague held political views ideologically opposed to yours? As social tensions rise due to polarizing political issues, even the smallest gestures can become subtle reflections of interpersonal relationships (Alesina & Tabellini, 2024; Nasuto & Rowe, 2024; Van Doesum et al., 2016a, 2016b).

People often engage in thoughtful gestures, such as leaving the last chocolate chip cookie, by considering others’ desires and needs when making decisions, a concept known as social mindfulness (Kirkland et al., 2022; Lemmers-Jansen et al., 2018; Van Lange & Van Doesum, 2015). This form of low-cost cooperation allows individuals to preserve others’ autonomy, enabling them to leave versus remove options in interdependent situations (Van Doesum et al., 2013, 2016a). Social mindfulness does not only involve the skill to recognize others’ preferences but also a willingness to leave others with choices. While individual traits matter, this willingness is also shaped by group identity (Guo & You, 2024; Van Doesum et al., 2016b). Group identity refers to a sense of belonging to a social group defined by shared characteristics or traits, such as nationality or political affiliation (Ashforth & Mael, 1989; Dhami et al., 2018; Felfe et al., 2018; Li, 2020; Lock & Funk, 2016). Notably, shared group identity serves as a strong cue for cooperation, as individuals are generally more willing to cooperate with ingroup members than with outgroup members (Dorrough et al., 2015; Ellemers et al., 2013; Everett et al., 2015; Tajfel et al., 1971; Yamagishi & Mifune, 2009; Zuo et al., 2018). This tendency to ingroup favoritism is particularly evident in economic games (e.g., dictator game, prisoner’s dilemma) that involve interdependent situations with a single choice, where individuals cannot benefit from reciprocating one another’s choice (Balliet et al., 2014). Social mindfulness adheres to a similar structure, relying on single cooperative choices with limited concerns for reciprocity (Van Doesum et al., 2013; Van Lange & Van Doesum, 2015). However, while economic games typically motivate cooperation through tangible gains and losses (Stallen & Sanfey, 2013), social mindfulness is not conceptualized as cooperation toward a shared materialistic goal (Van Doesum et al., 2025). Instead, it is rooted in subtle other-oriented behavior whose value lies in immaterial benefits, functioning as a signal of benevolence and respect that fosters interpersonal relationships (Lemmers-Jansen et al., 2018).

Building on this principle, Van Doesum et al., (2016b) explored how group identity influences such low-cost cooperative behavior by using the social mindfulness (SoMi; Van Doesum et al., 2013) paradigm among football teammates and rival team members. In this dyadic task, participants selected one common object (e.g., a hat) from a set of four, in which there were three identical (e.g., green hats) and one was unique (e.g., a red hat). Participants were informed that their choice would affect their interaction partner, who would subsequently choose from the remaining objects. Selecting one of the identical items throughout the task was deemed socially mindful. Participants were aware that the task was hypothetical; they would not receive any of the selected objects, nor would their interaction partner. The results revealed that, despite the absence of material gain, participants were significantly more likely to make socially mindful decisions when the interaction partner was a teammate rather than a rival. These findings highlight the influence of shared group identity in fostering cooperative behavior, demonstrating that even minimal costs can encourage individuals to act in the interest of their group.

While behavioral research shows that people tend to favor ingroup members in cooperative situations, neuroimaging studies reveal how group identity influences brain activity during social decision-making (Hein et al., 2010; Izuma & Adolphs, 2013; Xu et al., 2009). For instance, performing a prisoner’s dilemma with ingroup members as opposed to outgroup members, heightened activation in the default mode network (DMN), a network associated with self-referential processes and understanding other’s mental state (i.e., mentalizing; Menon, 2023; Rilling et al., 2008). In contrast, interactions with outgroup members tends to recruit the frontoparietal network (FPN) more strongly, reflecting heightened demands on cognitive control, outcome monitoring, and the integration of complex information (Badre & Esposito, 2007; Berry et al., 2017; Corbetta & Shulman, 2002; Vincent et al., 2008). Interestingly, using the SoMi paradigm without disclosing the interaction partner’s group identity, Lemmers-Jansen et al. (2018) also found heightened activation from regions of both the DMN and FPN, along with reward-related areas. Particularly, socially mindful decision-making was related to increased activation within the anterior cingulate cortex (ACC) and precuneus, regions of the FPN, and within the medial prefrontal cortex (mPFC), posterior cingulate cortex (PCC), and temporoparietal junction (TPJ), core regions of the DMN. These initial insights may suggest that social mindfulness is associated with mentalizing processes mediated by the DMN. Additionally, being socially mindful may require integrating external cues with internal goals, processes associated with the FPN, to assess the impact of one’s decisions on their interaction partner’s outcomes (Spreng et al., 2010). Finally, the activation of reward-related regions (i.e., caudate) aligns with previous studies implying that prosocial behavior is intrinsically rewarding (Cutler & Campbell-Meiklejohn, 2019; Declerck et al., 2013; Lemmers-Jansen et al., 2019; Luo, 2018; Walsh et al., 2021). Nonetheless, since the interaction partner’s group identity remained unclassified throughout the SoMi paradigm, the extent to which this interpersonal factor influences the engagement of these neural networks in socially mindful decision-making is still unknown.

Therefore, the current functional magnetic resonance imaging (fMRI) study investigates how group identity influences socially mindful behavior and its neural underpinnings. In research, group identity is often manipulated using minimal and arbitrary cues, such as assigning a specific color, which carry no inherent meaning (Dunham, 2018; Iani et al., 2011; Tajfel & Billic, 1974). Instead, this study incorporates a real-world context by focusing on refugee migration, a highly polarizing social issue that has created sharp ideological and social divides over the past decade (Bock, 2018; Dancygier & Margalit, 2020; Glinitzer et al., 2021; Lim & Ahn, 2024). Consequently, the SoMi paradigm will be employed among individuals with strong pro- or anti-refugee stances, introducing a setting where participants are likely to have biases toward certain interaction partners and reduced motivation to display socially mindful behavior (Van Doesum et al., 2016b). Throughout the study, it is hypothesized that people make more socially mindful decisions when interacting with an ingroup member compared to an outgroup member or unclassified other. At the neural level, when group identity information is absent, socially mindful decisions are hypothesized to heighten brain activity in areas associated with social decision-making, mentalizing and reward processes (i.e., FPN, DMN, caudate). However, when group identity is introduced, socially mindful decision-making for ingroup members, compared with outgroup members and unclassified others, is hypothesized to heighten activity in brain areas associated with mentalizing (i.e., DMN). Finally, it is hypothesized that making socially mindful decisions for outgroup members, compared with ingroup members and unclassified other, increases activity in brain areas associated with cognitive control (i.e., FPN).

## Methods

### Preregistration

The study’s research questions, hypotheses, and fMRI data analysis plan are preregistered at: 10.17605/OSF.IO/WDHYJ. Related codes used for quality controls and the preprocessing of the fMRI data are available at: https://github.com/marietvbuuren/self_other_2020/tree/master/preprocessing_and_analyses, though combining of the echoes is missing. Because the participants did not give consent, the data cannot be publicly shared.

### Participants

Sixty-one neurotypical adults were recruited using online platforms (e.g., Facebook, Link2trials) or were approached in person at Dutch universities or shopping areas. Recruitment involved a two-stage procedure: potential participants first completed the Refugee Scale online to assess their stance on refugee migration. Eligible participants were then invited and retested at the research facility to confirm consistency. Ultimately, the study included fluently Dutch-speaking participants aged 18 or older with strong stances on refugee migration. Participants were excluded if they had any contradictions for MRI scanning or clinically diagnosed psychological disorder(s). Eventually, 16 of the 61 included participants were excluded from the analysis. Specifically, four participants provided extreme scores (only mindful or unmindful responses) throughout the experiment, one participant shifted in political stance between the screening and retest, for two individuals it was impossible to combine the echoes, two participants had missing trials, and four participants did not meet the mindful trial cutoff of eight. With a minimum of nine trials per condition, the trial count was sufficient to ensure adequate power (Vizioli et al., 2018). Additionally, screening data from the Refugee Scale were incomplete for five participants. For these individuals, retest scores of the Refugee Scale and manipulation check responses from the scanning day were used for categorization. Based on these data, two participants were excluded as their scores represented neutral attitudes toward refugees and one participant was excluded due to substantial inconsistency between the Refugee Scale and manipulation check answers. Eventually, data from 45 participants (22 females, *M*_age_ = 23.64, standard deviation [*SD*] = 5.33) were included in the analyses to test the study’s hypotheses. This sample size is considered sufficient to detect brain regions associated with large effect sizes and exceeds the participants numbers typically reported in fMRI studies (Geuter et al., 2018; Poldrack et al., 2017; Szucs & Ioannidis, 2020).

### Procedure

This research was approved by the Scientific and Ethical Review Board of the Vrije Universiteit Amsterdam. After signing the informed consent, participants completed the Refugee Scale online at home via Qualtrics software (Qualtrics, 2019) to assess their stance on refugee migration. Those classified as pro- or anti-refugee based on their scores were invited to the scan session.

Upon arrival, participants were informed about the scanning procedure and completed the Refugee Scale again, allowing the experimenter to assess the alignment or opposition of political stance throughout the task. Each scan session started with a pre-scan and a brief survey scan, followed by a 7-minute structural scan. During the structural scan, instructions of the SoMi task were presented on the screen. These instructions highlighted that the task involved interacting with a partner and required the participants to carefully think about not only which of the presented objects they preferred and would hypothetically take home, but also which object their interaction partner would get to take home. Additionally, participants were informed that their choice would impact this partner, who would select from the remaining objects, as the collection of objects would not be replenished.

After the structural scan, the participants performed three rounds of the SoMi task. To investigate how group identity affects the behavioral and neural correlates of social mindfulness, identity-related information about the interaction partner was provided before each round. In the first round, no such information was provided, and participants interacted with an unclassified interaction partner. In the second and third round, however, participants were informed whether their interaction partner shared their stance on refugee migration (ingroup) or held opposing views (outgroup). This information, manufactured by the researchers for the purpose of the experiment, was conveyed by displaying the partner’s pro- or anti-refugee responses on the Refugee Scale. This manipulation was designed to induce a sense of group identity throughout the second and third round. The order of the ingroup and outgroup rounds was counterbalanced across participants.

The entire scan session lasted approximately 40 min. Afterwards, participants completed an exit survey and were debriefed about the experiment. Each participant received €25 in compensation and travel expenses were reimbursed.

#### Stance on refugee migration

To manipulate group identity, participants were preselected on their stance on refugee migration using the Refugee Scale (Table 1). This scale contains six statements on refugees that were to be rated on a Likert scale from 1 (“completely disagree”) to 7 (“completely agree”), with total scores ranging from a minimum of six to a maximum of 42 points. The scale showed a good internal consistency (*α* = 0.86). Participants were classified as pro-refugee if their score was below 17 points and as anti-refugee if their score exceeded 27 points. On the 7-point Likert scale, scores ≤ 16 (mean ≤ 2.67) indicate consistently positive attitudes, while scores ≥ 27 (mean ≥ 4.5) reflect moderately to highly negative attitudes (Pederson et al., 2005). By excluding individuals with neutral attitudes, this cutoff ensured that the final sample included only participants with clearly positive or negative attitudes, highlighting meaningful differences in stance toward refugees. The scale was administered in Dutch and incorporated into the SoMi paradigm to induce a sense of group identity throughout the study (see *Procedure*).

**Table 1:** The refugee scale

Item 1	Refugees receive more from the Netherlands than they contribute to the Netherlands
Item 2	The inclusion of refugees leads to higher taxes for Dutch citizens
Item 3	Refugees pose a threat to the job security of Dutch citizens
Item 4	The influx of refugees enriches Dutch culture.*
Item 5	The (moral and religious) values and beliefs of refugees are in line with that of most Dutch people.*
Item 6	Refugees must adapt to and accept the Dutch way of life

The English translation of the Dutch version of the Refugee Scale measuring participant’s stance on refugee migration. *Reverse-scored items

#### Manipulation checks

To ensure that participants accurately perceived the manipulation of group identity per round, manipulation check questions were included in the exit survey. These questions assessed the participants’ sense of connectedness (“Do you feel connected to player 1/2/3?”), similarity (“Do you think you have many similarities with player 1/2/3?”) and liking (“Do you like player 1/2/3?”) for each interaction partner presented throughout the SoMi paradigm. All responses were rated on a Likert scale ranging from 1 (“Very Much Not”) to 7 (“Very Much”), with higher scores reflecting stronger positive sentiment.

### Measures

#### Social mindfulness

To measure social mindfulness, participants performed the SoMi paradigm (Van Doesum et al., 2013) while fMRI data were collected. Each participant completed three rounds of the task, with each round lasting eight minutes and consisting of two trial types presented in a quasi-randomized order: experimental and control. Each round began and concluded with a 6000 ms waiting period. Totally, the duration of the task was 24 min.

**Fig. 1:**
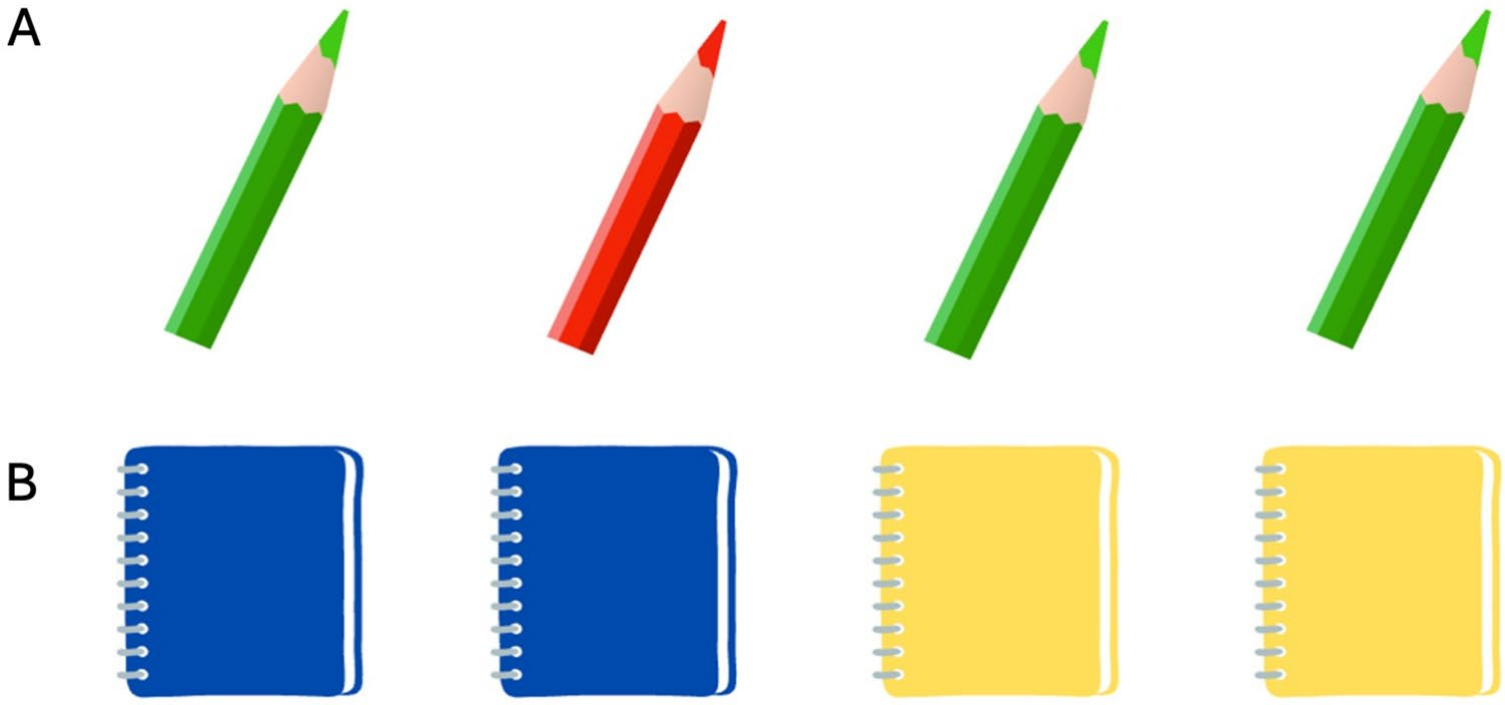
Example of the SoMi paradigm. **A.** Experimental trial. **B.** Control trial

In the experimental trials, a set of four objects was displayed in a 3:1 ratio for 3000 ms (Fig. 1A). Per trial, the objects came from different categories (e.g., pencils, booklets). Once the objects appeared on the screen, participants could select one of the objects using the index and middle finger of both hands by pressing the button corresponding to an object (e.g., the left middle finger for the leftmost object). After selecting an object, the choice was immediately highlighted and displayed for the remainder of the trial duration, followed by a jittered interstimulus interval of 0, 1000, or 2000 ms. If the participant missed a trial, a message of encouragement was given for 4000 ms. Selecting one of the identical items was categorized as a socially mindful decision, while choosing the unique item was considered socially unmindful. Each round contained 30 experimental trials**.**

Control trials were visually aligned to the experimental trials and adhered a similar procedure. However, no unique items were shown during these trials, as the objects were presented in a 2:2 ratio (Fig. 1B). Therefore, no decision could limit the options available to the participant’s interaction partner. Each round contained 30 control trials.

Twenty rest periods were incorporated throughout each round, during which participants viewed an asterisk at the center of the screen for 3000 ms and were instructed not to respond.

### MRI data acquisition

The brain imaging data were collected at the Spinoza Centre for Neuroimaging, using a 3.0 Tesla Philips Ingenia MRI scanner equipped with a 32-channel-phase head coil (Philips Healthcare, Best, The Netherlands). During the SoMi task, the 214 functional images were obtained using a T2*-weighted EPI multi-echo sequence (TR = 2.375 s; TE = 9.0, 26.4, 43.8 ms; FA = 76.1°; FOV = 224 mm; voxel size 3 × 3 × 3 mm; 37 slices). Structural images were collected for anatomical reference using a T1-weighted (TR = 8.3; TE = 3.8; FA = 8°; matrix = 240 × 188 mm; FOV = 240 × 188x220; voxel size 1 × 1 × 1 mm; 220 slices in total).

### fMRI data preprocessing

The multiecho data were merged with MATLAB 2019a (MathWorks). First, the initial echo image of every scan was aligned with the first echo of the first scan. The realignment parameters obtained from this step were subsequently applied to all other echoes. Then, the contrast-to-noise ratio for each echo was calculated using the 30 prescans to determine the optimal weighting of the three echo times for every voxel. Next, these weighting parameters were applied to combine the three echo images of each scan into a single image. The generated images were preprocessed with SPM12 software (https://www.fil.ion.ucl.ac.uk/spm/software/spm12/). Initially, the functional images were slice time corrected with the temporal middle slice (slice 19) serving as the reference and a co-registration was applied between the structural and mean functional image. This was followed by a unified segmentation, where the parameters derived from the segmentation process were applied to normalize both the functional and structural data to Montreal Neurological Institute (MNI) stereotaxic space. Lastly, the normalized functional data were smoothened using a 6 × 6 × 6 full-width half-maximum (FWHM) Gaussian smoothing kernel.

### Data analysis

#### fMRI data analysis

The preprocessed functional images for all three runs (i.e., unclassified, ingroup, outgroup) were analyzed using a General Linear Model (GLM) in SPM12. At the first level, the regressors of interest for each run included the mindful experimental trials, unmindful experimental trials, and the control trials with an onset set at the beginning of each respective trial. The events were modelled to have a duration corresponding to the reaction time, with a maximum of 3000 ms. Furthermore, a regressor of no-interest was added to model the missed trials (duration of 3000 ms), the brief period at the start of the task (duration of 6000 ms), and the encouragement trials following a missed trial (duration of 4000 ms). A boxcar functional with a canonical Hemodynamic Response Function (HRF; Friston et al., 1994) was used to model all regressors. Finally, six motion regressors were incorporated into the model and a high-pass filter with a cutoff of 128 s was used to remove low frequencies. Subsequently, contrast images for each participant were defined to compare the socially mindful decisions for ingroup, outgroup, and unclassified runs of the experimental trials with control trials of the same run. Additionally, socially mindful decisions and unmindful decisions were contrasted against baseline.

The resulting contrast images for each participant were included in a second-level analysis to identify whole-brain task-related fMRI signals. In line with the research aim, the analysis focused solely on socially mindful trials. Additionally, by comparing mindful trials relative to control trials, rather than to unmindful trials, the study ensured enough power as some participants had only a small number of unmindful trials. First, a one sample *t*-test was conducted to investigate the activation pattern related to socially mindful decision-making without disclosing the interaction partner’s group identity ([Mindful_Unclassified > Control_Unclassified]). Second, a paired-sample *t*-test was used to determine the influence of interacting with an ingroup member on socially mindful decisions, comparing [Mindful_Ingroup > Control_Ingroup vs. Mindful_Outgroup > Control_Outgroup] and [Mindful_Ingroup > Control_Ingroup vs. Mindful_Unclassified > Control_Unclassified]. Finally, to investigate the influence of interacting with an outgroup member on socially mindful decisions, another paired-sample *t*-test was performed for the contrasts [Mindful_Outgroup > Control_Outgroup vs. Mindful_Ingroup > Control_Ingroup] and [Mindful_Outgroup > Control_Outgroup vs. Mindful_Unclassified > Control_Unclassified]. The results were evaluated for significance using cluster-based inference, with a cluster-defining threshold of *p* <.001 and a family-wise error corrected (FWE) cluster probability of *p* <.05. For each contrast, the corrected cluster size thresholds were computed using the Random Field Theory-based SPM extension “CorrClusTh.m v1.12” (Nichols & Wilke, 2012).

#### Behavioral data analysis

IBM SPSS statistics (Version 29) was used to analyze the demographic and behavioral data. A repeated-measures ANOVA with recipient as within-group factor was performed to investigate the differences in socially mindful decision-making across the three interaction partners, followed by paired-samples t-tests to assess the frequency of socially mindful decisions and differences between conditions. A *p*-value of.05 was used as a threshold for statistical significance, with Bonferroni correction applied for multiple comparisons.

## Results

### Behavioral results

#### Manipulation checks

The participant characteristics are displayed in Table 2. A repeated measures ANOVA revealed a significant difference in liking across the three interaction partners *F*(2, 44) = 13.52, *p* <.001, *η*^*2*^*ₚ* =.235. Post-hoc analysis showed that participants liked ingroup interaction partners (*M* = 4.96, *SD* = 1.35) significantly more than outgroup members (*M* = 3.67, *SD* = 1.21), *MD* = 1.29, 95% CI [.55, 2.03], *p* <.001, *d* = 0.65. Also, unclassified interaction partners (*M* = 4.62, *SD* =.86) were liked more than outgroup members, *MD* =.96, 95% CI [.385, 1.53], *p* <.001, *d* = 0.62. No significant difference in liking was found between ingroup and unclassified interaction partners (*p* =.526).

The level of connectedness was significantly affected by the interaction partners’ group identity, *F*(2, 44) = 10.80, *p* <.001, *η*^*2*^_*p*_ =.197. Post-hoc testing revealed that participants felt significantly more connected to an ingroup interaction partner (*M* = 4.36, *SD* = 1.79) compared with an outgroup interaction partner (*M* = 2.84, *SD* = 1.58), *MD* = 1.51, 95% CI [.65, 2.37], *p* <.001, *d* = 0.65. Additionally, participants felt more connected to the unclassified interaction partner (*M* = 3.69, *SD* = 1.61) than to outgroup members, *MD* =.84, 95% CI [.09, 1.60], *p* =.024, *d* = 0.42. No significant differences were found between the ingroup and unclassified interaction partners (*p* = 0.146).

Mauchly’s test indicated that the assumption of sphericity was violated, *χ*^*2*^(2) = 7.60, *p* =.022, so the Greenhouse–Geisser corrected test is reported (*ε* =.861). The results revealed a main effect of group identity on ratings of similarity, *F*(1.72, 44) = 14.05, *p* <.001, *η*^*2*^_*p*_ =.242. Post-hoc comparisons indicated that participants felt significantly more similar to ingroup (*M* = 4.76, *SD* = 1.90) than to outgroup interaction partners (*M* = 3.02, *SD* = 1.56), *MD* = 1.73, 95% CI [.70, 2.76], *p* <.001, *d* = 0.63). Participants also felt more similar to unclassified others (*M* = 4.60, *SD* = 1.23) than to outgroup interaction partners, *MD* = 1.58, 95% CI [.87, 2.29], *p* <.001, *d* = 0.83. No significant difference in similarity was found between ingroup and unclassified interaction partners, Bonferroni-corrected *p* = 1.000.

**Table 2:** Participant characteristics

Participants, *N*	45
Age, mean (*SD*), range	23.64 (5.33), 18–41
Gender, female *N* (%)	22 (49%)
Handedness, right-handed (%)	37 (82%)
View on refugees, pro/anti *N* (%) Gender within pro/anti, female *N* (%)	26 (58%)/19 (42%) 13 (50%)/9 (47%)

#### Behavioral analyses

**Fig. 2:**
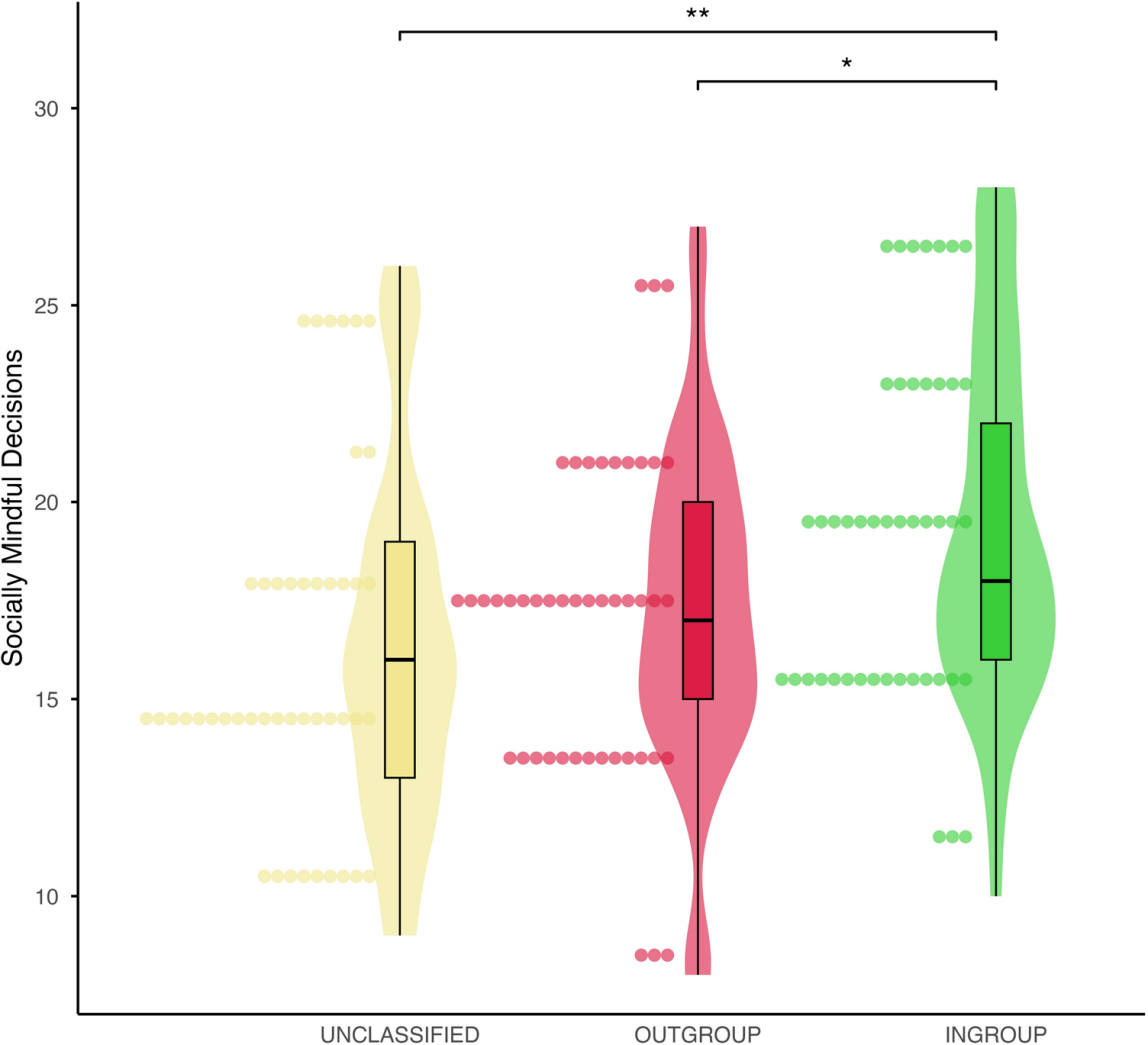
Distribution of socially mindful decisions by interaction partner during the SoMi task. *p <.05, **p <.001

A repeated measures ANOVA was conducted to compare responses across the three interaction partners. Mauchly’s test indicated that the assumption of sphericity was met, χ^2^(2) = 4.98, *p* =.083. The results revealed a significant effect of interaction partner on socially mindful decisions, *F*(2, 44) = 7.76, *p* =  <.001, *η*^*2*^*ₚ* =.150. Follow-up paired-samples t-test showed that participants made signficantly more socially mindful decisions when performing the SoMi paradigm with an ingroup member (*M* = 19.36, *SE* =.67) compared with an outgroup member (*M* = 17.18, *SE* =.61), *MD* = 2.18, 95% CI [.36, 3.99], *t*(44) = 2.42, *p* =.010, *d* = 0.36. Similarly, participants made significantly more socially mindful decisions when interacting with an ingroup member than with an unclassified other (*M* = 16.31, *SE* =.67), *MD* = 3.04, 95% CI [1.70, 4.39], *t*(44) = 4.55, *p* <.001, *d* = 0.68. In contrast, participants did not make more socially mindful decisions when interacting with an outgroup member compared with an unclassified other, which aligns with implicit expectations (*MD* =.87, 95% CI [−.75, 2.48], *t*(44) = 1.08, *p* =.143). Figure 2 displays the distribution of the socially mindful responses per interaction partner type.

### fMRI results

All results are reported at a cluster-defining threshold of *p* <.001 and a *p* <.05 FWE-corrected cluster threshold.

**Fig. 3:**
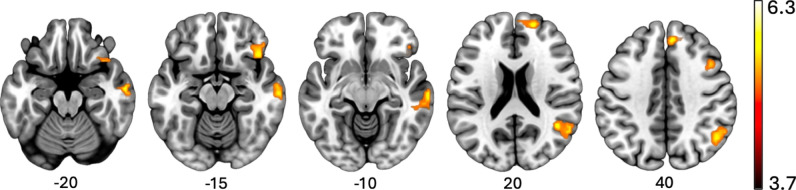
Whole-brain analysis results for socially mindful decisions for unclassified interaction partners. Results are shown at a cluster-defining threshold of *p* < .001 and a *p* < .05 FWE-corrected cluster threshold. The numbers in the figure represent z-coordinates. The color bars indicate *t*-values

Initially, the neural activity related to socially mindful decision-making was examined by contrasting the socially mindful trials with the control trials during interactions with an unclassified partner. The whole-brain analysis results revealed that making socially mindful decisions activated regions of the DMN, especially the rTPJ and right superior frontal gyrus (dorsal mPFC). Notably, activation in the dorsal mPFC extended into the dorsolateral prefrontal cortex (dlPFC), a region included in the FPN. Furthermore, activations were observed in the right middle temporal gyrus (MTG) and right lateral orbitofrontal gyrus (OFC) (Table 3A; Fig. 3).

**Table 3:** Whole-brain analyses results on socially mindful choices, socially mindful decision-making for outgroup members versus ingroup members, and socially mindful decision-making for outgroup members versus unclassified interaction partners

Brain region and contrast	MNI coordinates			Z score	voxels
	x	y	z		
**A. Mindful > Control**					
R middle temporal gyrus (MTG)	51	− 39	− 6	5.21	197
R Superior frontal gyrus (mPFC)	9	39	48	5.00	292
R Angular gyrus (TPJ)	54	− 57	30	4.71	274
R Superior frontal gyrus (mPFC)	18	54	21	4.55	55
R Lateral orbitofrontal gyrus (OFC)	45	24	− 15	4.30	53
**B. Mindful outgroup >** **Mindful ingroup**					
R Anterior insula (AI)	33	12	− 3	4.26	49
**C. Mindful outgroup >** **Mindful unclassified**					
L superior frontal gyrus (dACC)	−6	24	42	4.83	92

MNI coordinates indicate the peak voxels of the first local maximum within each cluster. Task-specific contrasts are presented in bold. **A.** Cluster-defining threshold of* p* <.001 and a *p* <.05 FWE-corrected critical cluster size of 42 was applied **B.** Cluster-defining threshold of* p* <.001 and a *p* <.05 FWE-corrected critical cluster size of 47 was applied. **C.** Cluster-defining threshold of* p* <.001 and a *p* <.05 FWE-corrected critical cluster size of 48 was applied

**Fig. 4:**
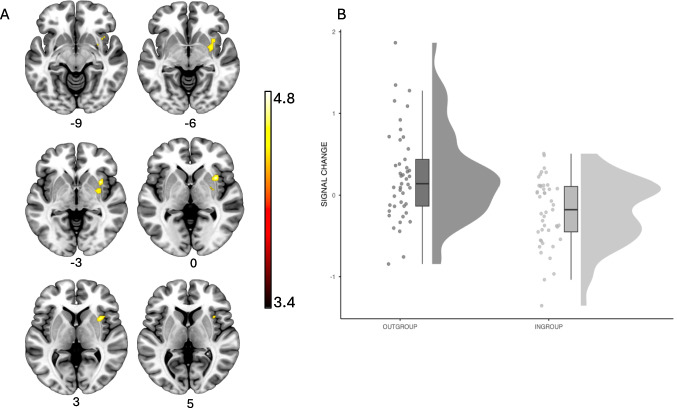
Whole-brain analysis results for socially mindful decisions for outgroup members compared to ingroup members. **A.** Increased activation in the AI during socially mindful decision-making for outgroup versus ingroup interaction partners. The numbers in the figure represent z-coordinates. Results are shown at a cluster-defining threshold of *p* < .001 and a *p* < .05 FWE-corrected cluster threshold. The color bars indicate *t*-values. **B.** For visualization, the signal extracted from the AI for outgroup and ingroup interactions partners

**Fig. 5:**
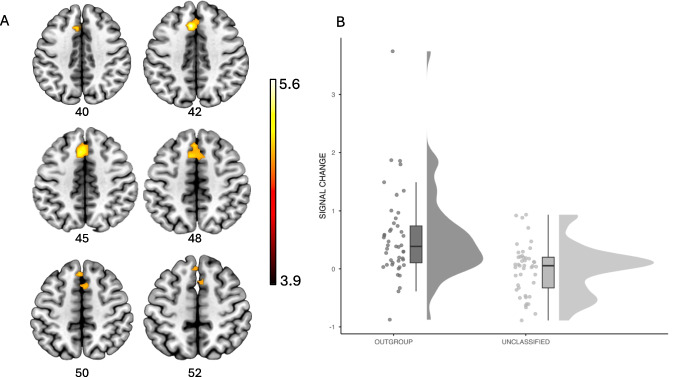
Whole-brain analysis results for socially mindful decisions for outgroup members compared to unclassified others. **A.** Increased activation in the dACC during socially mindful decision-making for outgroup versus unclassified interaction partners. The numbers in the figure represent z-coordinates. Results are shown at a cluster-defining threshold of *p* < .001 and a *p* < .05 FWE-corrected cluster threshold. The color bars indicate *t*-values. **B.** For visualization, the signal extracted from the dACC for outgroup and unclassified interactions partners

Subsequently, the influence of the interaction partners' group identity on the neural activation pattern during socially mindful trials was investigated. When comparing socially mindful trials for ingroup members to socially mindful trials for outgroup members or unclassified others no significant clusters were found. However, the whole-brain results did reveal that making socially mindful decisions for outgroup members compared with ingroup members, elicited heightened activation within the anterior insula (AI; Table 3B; Fig. 4A). Finally, the results showed that making mindful decisions for outgroup members, contrasted with unclassified others, engaged the left dorsal ACC (dACC; Table 3C; Fig. 5A). For visualization, contrast values were extracted from the AI cluster for each subject and averaged separately for the outgroup and ingroup interaction partner (Fig. 4B). The same procedure was performed for the dACC cluster for both the outgroup member and unclassified interaction partner (Fig. 5B).

### Exploratory post-hoc analyses

#### Influence of interaction order on socially mindful decision-making

Repeated-measures ANOVA showed no significant differences in socially mindful decision-making toward ingroup and outgroup interactions as a function of order (see Supplement 1).

#### DACC activation and self-reported connectedness

A Spearman correlation analysis showed no significant correlation between dACC activation and outgroup connectedness. Similarly, no significant correlation was found between dACC activation during socially mindful decisions involving unclassified interactions partners and connectedness ratings (see Supplement 2).

#### Influence of group identity on the neural correlates of socially unmindful decision-making

Finally, post-hoc whole-brain analyses were conducted to examine the neural activation patterns associated with socially unmindful decision-making (see Supplement 3). Applying the same minimal trial requirement for unmindful decisions, 18 participants from the original sample were excluded, leaving a small sample size of 27 participants (see Supplementary Table 1). All results were tested for significance using cluster-inference with a cluster-defining threshold of *p* <.001, and a cluster-probability of *p* <.05 FWE corrected cluster threshold. The results for socially unmindful decision-making revealed heightened activation in the AI, inferior frontal gyrus, superior frontal gyrus (dorsal mPFC), and lingual gyrus (see Supplementary Table 2 A). Also, findings showed that making unmindful decisions for outgroup members compared with ingroup members showed heightened activation in the middle frontal gyrus, dlPFC, and precuneus (see Supplementary Table 2B). Lastly, socially unmindful decisions toward outgroup members relative to unclassified others evoked heightened activation in the middle frontal gyrus and precuneus (see Supplementary Table 2 C). Given the relatively small sample size and limited statisical power, these findings should be interpretated with caution owing to the increased risk of false-positive results.

## Discussion

The current study investigated how group identity influences socially mindful behavior and its neural underpinnings, using the SoMi paradigm among individuals with strong pro- or anti-refugee stances. Consistent with our first hypothesis, individuals made more socially mindful decisions when interacting with ingroup members compared to outgroup members or unclassified partners. This behavior did not differ between interactions with outgroup members and unclassified others. Supporting our second hypothesis, socially mindful decisions made for unclassified others, engaged a region of the FPN (dlPFC) and regions of the DMN (dorsal mPFC and rTPJ), MTG and OFC, a region implicated in reward processing (Du et al., 2020; Rolls et al., 2020). Contrary to the expectations, no significant activation patterns during socially mindful decisions were found when comparing ingroup interaction partners with outgroup or unclassified interaction partners. Beyond the predefined hypotheses, socially mindful decision-making for outgroup interaction partners, compared with ingroup interaction partners, elicited heightened activation in the right AI. Finally, in accordance with our last hypothesis, decisions for outgroup interaction partners, as opposed to unclassified interaction partners, was positively associated with the dACC, a key node of the FPN previously linked to cognitive control (Alexander & Brown, 2015; Spreng et al., 2010).

The study’s behavioral findings demonstrate that shared group identity functions as a cue that promotes social mindfulness. Extending research on cooperation in economic games (Balliet et al., 2014), these results suggest that, even in low-cost and single choice interdependent scenarios, individuals tend to act in ways that benefit their ingroup. These findings coincide with previous research by Van Doesum et al., (2016b), which also observed ingroup favoritism among football teammates and rivals using the SoMi paradigm. Ingroup favoritism is commonly explained by two broad perspectives. One perspective argues that differences in social identity motivates such favoritism as the self is more strongly connected to the ingroup than the outgroup (Tajfel & Turner, 1979). The other perspective attributes this favoritism to the expectation that fellow group members are more likely to reciprocate favorable behavior, either directly or indirectly (Everett et al., 2015; Yamagishi et al., 1999). Even in scenarios where interactions are somewhat limited, differences in identity and distrust can be key to intergroup processes. To illustrate, researchers have proposed that outgroup members, especially those with opposing political ideologies, are often seen as being different from oneself, are liked and trusted less, with their beliefs being viewed as debatable at best (Finkel et al., 2020). These explanations are also consistent with a growing body of research on affective polarization in political views (Druckman et al., 2021).

The neural findings reveal increased activation in the rTPJ and dorsal mPFC, regions of the DMN, during socially mindful decisions involving unclassified interaction partners. This pattern aligns with social cognitive processes associated with the DMN, including the ability to infer others’ mental states and recognize how these can differ from one’s own (Amodio & Frith, 2006; Bitsch et al., 2018; Labutina et al., 2024; Menon, 2023; Salehinejad et al., 2021; Schurz et al., 2014, 2017; 2021; Van Den Bos et al., 2009). Interestingly, exploratory post-hoc whole-brain analysis showed that socially unmindful decisions toward unclassified interaction partners also engaged the mPFC, suggesting a potentially broader role of the DMN in supporting the mentalizing demands of the SoMi paradigm irrespective of decision type (Lemmers-Jansen et al., 2018).

Besides the DMN activation, heightened activity was observed during socially mindful decision-making in the MTG, a region involved in recognizing social cues and relaying this information to higher-order mentalizing areas, like the mPFC and TPJ (Gan et al., 2024; Jauniaux et al., 2019; Moriguchi et al., 2006; Rodrigo et al., 2014; Sugiura et al., 2013; Yu et al., 2020). Furthermore, socially mindful decisions may involve integrating external available information with internal representations, reflected by the observed activation patterns of the dlPFC and OFC (Lemmers-Jansen et al., 2018; Vincent et al., 2008). The dlPFC is closely associated with cognitive control functions, particularly in the coordination of goal-directed behavior and supporting adherence to social norms during interpersonal interactions (Ridderinkhof et al., 2003; Stallen & Sanfey, 2013; Webler et al., 2022; Yoder & Decety, 2020). Similarly, prior studies have found dlPFC activation patterns with DMN regions in both economic games and the SoMi paradigm (Baumgartner et al., 2011; Lemmers-Jansen et al., 2018; Schurz et al., 2021; Van Den Bos et al., 2009). These findings suggest that social decision-making involves more than just mentalizing, it also requires balancing self-interest with one’s emotional and social understanding of others (Báez-Mendoza et al., 2021; Labutina et al., 2024). Additionally, the OFC can guide social decision-making by generating value-based signals that integrate the reward-related outcomes with their associated hedonic qualities (Kringelbach, 2005a, 2005b; Rolls et al., 2020; Wallis, 2007). Given the intrinsically rewarding and socially valued nature of social mindfulness, the dlPFC could utilize the value representation of socially mindful behavior from the OFC to align internal goals with dominant social norms. Together, the activity of the dlPFC, OFC, and DMN may facilitate behavior that is both goal-directed and considerate of others’ autonomy of choice, reflecting a broader sensitivity to the social consequences of one’s actions and the ability to adjust them accordingly (Mischkowski & Glöckner, 2016).

Contrary to the expectations, no significant differences in neural activation were observed during socially mindful decisions involving ingroup members compared with outgroup or unclassified interaction partners. However, socially mindful decisions toward outgroup interaction partners, relative to ingroup interaction partners, elicited increased activation in the right AI. Although unanticipated, this finding aligns with prior research suggesting that this region is part of the salience network and is particularly responsive to outgroup-related cues (Falk et al., 2012; Kaplan et al., 2007; Menon & Uddin, 2010; Merritt et al., 2021; Shkurko, 2013; Touroutoglou et al., 2012). In addition, the right AI has been linked to both the integration of outgroup information for inferring interpersonal relationships and the experience of negative affect during aversive social interactions (Civai et al., 2012; Hein et al., 2010; Lau et al., 2020; Rilling et al., 2008; Tabibnia et al., 2008; Xu et al., 2009). Also, previous social decision-making research has associated right AI activation with interoceptive awareness of subjective emotional states and the detection of social norm violation (Bellucci et al., 2018; Craig, 2009; Feruglio et al., 2023; Krueger et al., 2020; Seara-Cardoso et al., 2016; Zichenko & Arsalidou, 2017). Accordingly, making socially mindful decisions toward outgroup members may (implicitly or explicitly) evoke a feeling of norm violation, thereby making such interactions more socially salient and emotionally significant than those with ingroup members.

Finally, socially mindful decisions toward outgroup members, compared with unclassified interaction partners, were associated with increased activity in the dACC. As part of the FPN, the dACC plays a central role in cognitive control, the ability to act in a deliberate and goal-directed manner while suppressing more automatic responses (Bognar et al., 2024; Boorman et al., 2013; Kolling et al., 2016; Ritz & Shenhav, 2024; Vincent et al., 2008). Specifically, the dACC is thought to monitor conflict between competing decision strategies, with heightened activation reflecting greater cognitive control demands when opposing tendencies are engaged (Botvinick et al., 2001; Dignath et al., 2020; Heilbronner & Hayden, 2016; Izuma & Adolphs, 2013; Pochon et al., 2008; Shenhav et al., 2014). In dyadic interactions, socially mindful behavior is often considered normative (Van Lange & Van Doesum, 2015). Consequently, deviations from such norms may elicit social disapproval, pressuring individuals to conform to socially desirable responses (Lanz et al., 2022; Van Doesum et al., 2013). Supporting this, multiple studies have implicated the dACC in detecting violations of social norms (Güroǧlu et al., 2010; Rilling & Sanfey, 2011; Schreuders et al., 2018). The observed increased dACC activation during socially mindful decisions toward outgroup members may therefore reflect the cognitive effort required to align behavior with these social norms while overcoming habitual outgroup biases and egocentric tendencies (Boorman et al., 2013; Dorrough et al., 2015; Fehr & Camerer, 2007; Hughes et al., 2017; Lockwood et al., 2022). Nonetheless, despite the broad agreement regarding the dACC’s involvement in cognitive control processes, our interpretation is speculative and future studies are needed to further disentangle the underlying cognitive processes of socially mindful behavior in social interactions.

While this study provides new insights into the neural underpinnings of socially mindful decision-making and how this is influenced by group identity, it has its limitations. First, the manipulation checks showed no significant difference in participants’ feelings of liking, connectedness, or similarity between ingroup members and unclassified others, suggesting that the group identity manipulation may not have had its intended effect (Chester & Lasko, 2021). Additionally, this could explain the absence of neural differences between ingroup members compared with outgroup or unclassified interaction partners during socially mindful decisions. Prior research indicates that neural representations of ingroup favoritism often depend on the perceived threat from outgroups (Molenberghs & Louis, 2018; Richins et al., 2018). Given that the participants were already familiarized with the Refugee Scale, future studies could consider using a more novel and more prominent political categorization marker. For example, incorporating political party logos or narrative descriptions that convey the interaction partner’s stance on refugee migration (Callahan & Ledgerwood, 2016; Chung & Slater, 2013). Still, the manipulation checks showed that outgroup members were perceived as intended and the behavioral results demonstrate an effect of group identity on socially mindful decision-making. Therefore, it is plausible that the manipulation checks may not fully reflect participants’ psychological state during the experiment (Hauser et al., 2018). Second, throughout the SoMi paradigm, each trial displays common objects from the same category that slightly differ in their characteristics. As a result, choosing the identical object can be motivated by personal preferences instead of social mindfulness (Van Doesum et al., 2025). However, the use of a diverse set of objects across randomized trials likely diminished the influence of individuals preferences by averaging out such effect.

## Conclusion

This study provides novel insights into the behavioral and neural processes involved in social mindfulness and how they are influenced by the group identity of an interaction partner. Consistent with prior research, individuals demonstrated more socially mindful behavior when they shared group identity with the other person. Notably, activation of the right AI may support the processing of the social and emotional significance of interactions with outgroup members, potentially arising from experienced norm violation. Furthermore, heightened activation of the dACC during outgroup interactions suggests that overcoming intergroup biases may require substantial cognitive effort, even in low-cost cooperative scenarios. These findings highlight the challenges of interpreting and adhering to social norms in everyday interactions when intergroup dynamics come into play. After all, leaving the last chocolate chip cookie might not be as effortless as it seems.

## Supplementary Information

Below is the link to the electronic supplementary material.

Supplementary file1 (DOCX 24.7 KB)

## Data Availability

The participants did not provide explicit consent for public archiving of the research data. Therefore, the data are not stored in a public repository. Anonymised data will be made available to individual researchers upon request, when compatible with the General Data Protection Regulation (GDPR).

## Code availability

The code used for quality controls and the preprocessing of the fMRI data (excluding the combining of the echoes) is available at: https://github.com/marietvbuuren/self_other_2020/tree/master/preprocessing_and_analyses

## Declarations

### Preregistration

The study’s research questions, hypotheses, and fMRI data analysis plan are preregistered at: 10.17605/OSF.IO/WDHYJ. All preregistered analyses are reported in the manuscript. In addition, post hoc exploratory analyses were conducted and are explicitly identified as exploratory.
