# Supplementary material for: Mindful minds: How group identity shapes brain and behavior in social decision-making
Source: Cogn Affect Behav Neurosci. 2026 Apr 17;26(4):1778–94. doi: 10.3758/s13415-026-01425-1 (PMC13385094; doi:10.3758/s13415-026-01425-1)
Supplement: Supplementary file 1 — Supplementary file1 (DOCX 24.7 KB) [file 13415_2026_1425_MOESM1_ESM.docx]

**Supplementary Material**

Mindful Minds: How Group Identity Shapes Brain and Behavior in Social Decision-Making

**Supplement 1**

*The Influence of Interaction Order on Socially Mindful Decision-Making*

A repeated measures ANOVA was used to test the differences in socially mindful behavior toward ingroup and outgroup interaction partners as a function of order (ingroup first vs. outgroup first). This analysis showed that the interaction between interaction partner type and order was not significant, *F*(1,44) = .000, *p* = .983, η² < .001, indicating that the order of interaction partner did not influence socially mindful behavior toward ingroup or outgroup partners. The main effect of order was also not significant, *F*(1,44) = .203, *p* = .655, *η²* = .005. Based on these results, we can conclude that interacting first with an ingroup or an outgroup partner did not affect behavioral outcomes.

**Supplement 2**

*DACC Activation and Self-Reported Connectedness*

An exploratory correlation analysis was performed to investigate the individuals dACC activation during socially mindful decision-making for outgroup members compared to ingroup interaction partners and self-reported measures of connectedness, As the data deviated from normality, (*D* = .164, *p* = 0.004 ), a Spearman rank-order correlation was conducted. The results showed no significant correlation between dACC activation and outgroup connectedness,

𝜌 = 0.197, *n* = 45, *p* = .194, indicating that heightened dACC activation was not significantly associated with the participants’ sense of connectedness with the outgroup. Similarly, no significant correlation was found between dACC activation during socially mindful decisions involving unclassified interactions partners and connectedness ratings,

𝜌 = -0.060, *n* = 45, *p* = .694.

**Supplement 3**

*The Influence of Group Idenity on the Neural Correlates of Socially Unmindful Decision-Making.*

For each participant, contrast images were computed comparing socially unmindful decisions in the ingroup, outgroup and unclassified runs with the corresponding control trials within the same run. These contrasts were then entered into a second-level analysis, and the same comparisons and statistical tests were applied as those used for the socially mindful trials. Firstly, a one-sample t-test was performed to examine the neural activation pattern related to socially unmindful decision-making [Unmindful_Unclassified> Control_Unclassified]. Secondly, a paired sample t-test was conducted to investigate the influence of interacting with an ingroup member on socially unmindful decisions, comparing [Unmindful_Ingroup > Control_Ingroup vs. Unmindful_Outgroup > Control_Outgroup] and [Unmindful_Ingroup > Control_Ingroup vs. Unmindful_Unclassified > Control_Unclassified]. Lastly, the influence of interacting with an outgroup member on socially unmindful decisions was investigated via another paired-sample t-test for contrasts [Unmindful_Outgroup > Control_Outgroup vs. Unmindful_Ingroup > Control_Ingroup] and [Unmindful_Outgroup > Control_Outgroup vs. Unmindful_Unclassified > Control_Unclassified]. The results were evaluated for significance using cluster-based inference, with a cluster-defining threshold of *p* < .001 and a family-wise error corrected (FWE) cluster probability of *p* < .05. The neural results for these comparisons are shown in Supplementary Table 2.

**Supplementary Table 1**

*Subsample Participant Characteristics*

| Participants, *N* |  | 27 |
| --- | --- | --- |
| Age, mean (*SD*), range |  | 23.04 (4.93), 18 – 35 |
| Gender, female *N* (%) |  | 12 (44%) |
| Handedness, right-handed (%) |  | 21 (78%) |
| View on Refugees, pro / anti *N* (%)  Gender within pro / anti, female *N* (%) |  | 15(55%) / 12 (45%)  6 (40%) / 6 (50%) |

**Supplementary Table 2**

*Whole Brain Analyses Results on Socially Unmindful Decision-Making, Socially Unmindful Decision-making for Outgroup Members Versus Unclassified Interaction Partners and Socially Unmindful Decision-Making for Outgroup Members Versus Ingroup members.*

| Brain region and contrast |  | MNI Coordinates | | |  | Z score | voxels |
| --- | --- | --- | --- | --- | --- | --- | --- |
|  |  | x | y | z |  |  |  |
| **A. Unmindful > Control** |  |  |  |  |  |  |  |
| L Anterior insula |  | -36 | 18 | -15 |  | 5.62 | 511 |
| R Orbitofrontal gyrus |  | 45 | 27 | -9 |  | 5.06 | 120 |
| R Superior frontal gyrus |  | 3 | 54 | 24 |  | 5.01 | 896 |
| R Lingual gyrus |  | 24 | -48 | -12 |  | 4.28 | 42 |
| **B. Unmindful outgroup >**  **Unmindful ingroup** |  |  |  |  |  |  |  |
| R Middle frontal gyrus |  | 36 | 57 | 15 |  | 4.43 | 59 |
| R Dorsolateral PFC |  | 30 | 3 | 39 |  | 4.32 | 92 |
| R Precuneus |  | 3 | -60 | 57 |  | 3.66 | 60 |
| **C. Unmindful outgroup > Unmindful unclassified** |  | |  |  |  |  |  |
| R Middle frontal gyrus |  | 42 | 15 | 48 |  | 4.06 | 66 |
| R Precuneus |  | 12 | -66 | 42 |  | 4.04 | 138 |

*Note.* MNI coordinates indicate the peak voxels of the first local maximum within each cluster. Task-specific contrasts are presented in bold. **A.** Cluster-defining threshold of *p* < .001 and a *p* <.05 FWE-corrected critical cluster size of 40 was applied **B.** Cluster-defining threshold of *p* < .001 and a *p* <.05 FWE-corrected critical cluster size of 43 was applied. **C.** Cluster-defining threshold of *p* < .001 and a *p* <.05 FWE-corrected critical cluster size of 43 was applied.
